# Supplementary material for: Natural History and Exploitation of the Harbor Porpoise (Phocoena phocoena Linnaeus, 1758) during the Neolithic (ca. 4000–2000 cal. BC) in the Eastern Baltic Region
Source: Animals (Basel). 2023 Mar 2;13(5):909. doi: 10.3390/ani13050909 (PMC10000223; doi:10.3390/ani13050909)
Supplement: Supplementary file 1 [file animals-13-00909-s001.zip › animals-2234783-supplementary.pdf]

animals

MDPI

Supplementary

Table S1. Data on vessels from Neolithic sites decorated with porpoise tooth impressions on the exterior.

Archaeological site (context), years of excavation, collections	No. of vessels (identified from rim sherds)	No. of vessels with porpoise tooth decoration on exterior	%	Notes
Sārnate (dwellings with Early Sārnate Ware), 1938-1940; 1949; 1953-1959 excavations. Material held at National History Museum of Latvia.	19	69	27.54	Published in Bērziņš 2008, Table 9, as "tooth stamp" decoration (the connection with porpoise had not yet been discovered) [41].
Siliņupe (area 7), 1988-1989 excavation. Material held at National History Museum of Latvia.	7	75	9.33	
Riņņukalns (vessels assigned to midden accumulation phase), 1874–1875, 1877, 1895, 1913, 1943-1944, 2011, 2017-2018 excavations. Material held at National History Museum of Latvia; Institute of History and Archaeology, Tartu University; temporarily stored at Institute of Latvian History, University of Latvia.	5	86	5.81	

Reference

41. Bērziņš, V. Sārnate: living by a coastal lake during the east Baltic Neolithic, Acta Universitatis Ouluensis B, Humaniora 86; Oulun Yliopisto: Oulu, Finland, 2008, Table 9.
